# Longitudinal Measurement Invariance of the Parenting Sense of Competence (PSoC): Evidence to Question Its Use?

**DOI:** 10.1111/cch.70030

**Published:** 2024-12-30

**Authors:** Nicole Gridley, Kate Mooney, Sarah Blower, G. J. Melendez‐Torres, Vashti Berry, Tracey Bywater

**Affiliations:** ^1^ Carnegie School of Education Leeds Beckett University England UK; ^2^ Department of Health Sciences University of York England UK; ^3^ Exeter Medical School University of Exeter England UK

**Keywords:** confirmatory factor analysis, infants, parenting sense of competence, parents

## Abstract

**Background:**

This study investigated the factor structure of the parenting sense of competence (PSoC), a measure of parenting self‐efficacy, in a sample of parents recruited when their infants were under 2 months old. Due to the lack of longitudinal analysis of the PSoC's factor structure over time, the study sought to establish if the published two‐factor structure was consistent over an 18‐month period.

**Methods:**

Data collected from 536 parents who had participated in a randomised controlled trial of universal proportionate parenting support, delivered in five sites in England, were subject to confirmatory factor analysis (CFA).

**Results:**

CFA revealed that a three‐factor model was the best fit for the data. Longitudinal measurement invariance testing examined the stability of the three‐factor model across an 18‐month period. The results suggest that while the PSoC appeared to have configural variance, the metric and scalar variance were not supported. PSoC may be unstable across time and might be unreliable as a measure of parenting competence in parents of infants.

**Conclusion:**

These findings are particularly salient for researchers and clinicians who are utilising the PSoC as a measure of change in routine practice or as part of evaluations of interventions. Further investigation of individual items is needed to refine the PSoC and improve its psychometric validity. Additional analyses are also needed to establish the invariance of the measure across different groups (age, sex, ethnicity and socioeconomic status).

**Summary**

- A three‐factor model of the 17‐item PSoC, consisting of the subscales *interest*, *satisfaction* and *self‐efficacy*, was shown to be more favourable to the original two‐factor model for parents of infants aged birth to 20 months.
- The three‐factor model shows acceptable levels of configural variance over an 18‐month period.
- The three‐factor model shows instability in metric and scalar invariance over an 18‐month period for parents of infants aged birth to 20 months.
- Further investigations of the longitudinal variance of the PSoC are needed to establish its utility as a measure of change for clinical practice and research.

## 1 Introduction

Self‐efficacy functions as a moderator of good quality parent–child relations, with caregivers who report lower levels of perceived control over their children's behaviour less able to cope effectively with difficult child behaviour (Jones and Prinz 2005). Parenting self‐efficacy is described as the belief that they (the parent) can identify and respond to their child's needs appropriately and is considered to fluctuate according to specific situations and tasks (Bandura 1997). In contrast, satisfaction in the parenting role is considered a more stable construct that is less likely to vary because of the context and situation (deMontigny and Lacharite 2005). Evidence suggests that both self‐efficacy and satisfaction are latent constructs of a broader sense of parental competence and psychological well‐being. Both are said to influence children's psychological adjustment and cognitive functioning via parenting practices and behaviours (Jones and Prinz 2005). Furthermore, research indicates that interventions aimed at increasing parenting self‐efficacy and satisfaction in the early years are effective at improving good quality parent–child interactions in the short‐ and long‐term (Bloomfield and Kendall 2012; Jones and Prinz 2005). Subsequently, reliable and valid measures of parenting competence may provide researchers and clinicians the opportunity to screen and identify issues early in the child's life so appropriate provisions for intervention can be made before problems become entrenched (Whittaker and Cowley 2012).

Since Bandura's seminal work, several self‐report measures of parenting self‐efficacy have been developed. In a review of parenting self‐efficacy, Jones and Prinz (2005) identified eight self‐report measures commonly adopted within the literature. Of these, the parenting sense of competence (PSoC; Gibaud‐Wallston and Wandersman 1978; Johnston and Mash 1989), a self‐report measure of parenting self‐efficacy and satisfaction originally designed for, but not initially validated on, first‐time mothers of very young infants, was identified as the most popular measure implemented by researchers. It contains 17 items organised into two subscales: efficacy, which includes parenting capabilities and problem‐solving abilities, and satisfaction, capturing parental anxiety, motivation and frustration. More recently, Blower et al. (2019) identified the PSoC as one of only three self‐report measures of parenting attitudes and beliefs commonly implemented as outcome measures in randomised controlled trial (RCT) evaluations of parenting programmes designed for parents of children under 6 years. However, despite its popularity, psychometric evidence (reliability and validity) to support its use across two distinct age groups (birth to 5 years and 5–18 years) has proven limited (Blower et al. 2019). Specifically, evidence of its structural validity has been questioned due, in part, to the limited number of validation studies that have been published and their reliance on cross‐sectional data (Hurley et al. 2014). Moreover, no validation has been conducted using a sample from the United Kingdom.

The PSoC was first described as part of a paper presented to the American Psychological Association (Gibaud‐Wallston and Wandersman 1978). However, there is no published record to indicate how the original scale was developed. To date, five studies have investigated the factor structure of the PSoC (Gilmore and Cuskelly 2009, 2024; Johnston and Mash 1989; Ohan, Leung, and Johnston 2000; Rogers and Matthews 2004). Of these, only three have provided evidence to support the original two‐factor model, and all studies have indicated item‐level discrepancies. Moreover, while the PSoC is frequently used with parents of children spanning a large age range (birth to 16 years), there has been no longitudinal analysis of the PSoC's factor structure over time within the same population.

The inconsistent findings are potentially concerning given the popularity of the PSoC's use in both research and practice. Published literature suggests that the original 17‐item PSoC has yet to be validated comprehensively with parents of infants—the precise population it was originally designed for. Furthermore, evidence to support the PSoC is solely dependent upon cross‐sectional data, and, therefore, the reliability of the PSoC's subscales over time cannot be assumed. Assessment of a measure's structural stability or invariance over time and across groups is paramount for determining whether a construct is being measured in the same way and confirms that observed similarities or differences in participant scores are meaningful (Vandenberg and Lance 2000). Therefore, the current study will provide the first longitudinal examination of the PSoC using data drawn from a sample of parents with infants. We ask the following:

1. Which factor model (two or three) best fits the data provided by a sample of parents living in England when their child was less than 2 months old?
2. Does the factor model (identified above) demonstrate longitudinal measurement invariance over an 18‐month period?

## 2 Method

### 2.1 Participants

Data from the external pilot and main trial phase of the Enhancing Social‐Emotional Health and Wellbeing in the Early Years (E‐SEE) study (ISRCTN11079129) was used. The RCT, assessing the efficacy and effectiveness of a proportionate universal parenting intervention, was not effective and showed no significant differences between intervention and control groups across any of the outcomes (Bywater et al. 2018, 2022). Data from both intervention and control participants are included in the current study.

Participants were mothers of infants aged 8 weeks old living across five geographical areas in the south, north and middle of England. Mothers were referred to the RCT by health visitors, family support workers or self‐referral. Eligibility criteria stated that participants had primary caregiving responsibility for an infant aged 8 weeks, were competent to give consent and were willing to participate in the research. Participants were not eligible if their infant had obvious or diagnosed organic or developmental difficulties.

For the present study, only participants with a complete set of 17 PSoC items were included. Hence, a total of 536 mothers were included in the testing of factor structure at baseline (RQ1). Sample demographics can be found in Table 1. A total of 46 participants were lost to follow‐up across the four timepoints. We opted for listwise deletion methods to deal with this missing data on the basis that, to have confidence in our conclusions, all analyses should be calculated using the same set of cases, and that methods for measurement invariance with imputed data are not well‐developed (Liu et al. 2017). Removing these cases resulted in 490 full cases for inclusion in the testing of the factor structure across 18 months (RQ2). Because there is no closed‐form power calculation for longitudinal measurement invariance analyses, we were unable to conduct a power calculation. However, most fit indices in structural equation modelling were developed for sample sizes of between 150 and 250, suggesting our sample would have been adequate to reject meaningful differences in measurement invariance.

**TABLE 1:** Descriptive statistics of the sample.

	Baseline sample (*n* = 536)
Parent ethnicity (%)	
English/Welsh/Scottish/Northern Irish/British	398 (74%)
Indian	38 (7%)
Pakistani	34 (6%)
Any other White background	33 (6%)
Other	25 (5%)
Missing	8 (1%)
Parent marital status (%)	
Married	341 (64%)
Cohabiting	132 (25%)
In a relationship	35 (7%)
Single	27 (50%)
Missing	1 (< 1%)
Parent highest qualification (%)	
Postdoctoral	10 (2%)
Masters	55 (10%)
A degree	164 (31%)
Certificate in higher education	57 (11%)
A, AS levels	39 (7%)
O levels	67 (12%)
Vocational	96 (18%)
Overseas	16 (3%)
None of the above	27 (54%)
Missing	5 (< 1%)
Child female (%)	271 (51%)
PSoC scores	
PSoC score T1 (*n* = 536)	81.35 (9.60)
PSoC score T2 (*n* = 490)*	83.05 (9.58)
PSoC score T3 (*n* = 490)*	83.91 (9.03)
PSoC score T4 (*n* = 490)*	82.78 (9.42)

* Including only participants with data at the first and last timepoints (to align with the sample in measurement invariance tests).

### 2.2 Procedure

Consenting participants were randomised to either the intervention or control arms (services as usual) at a 2.9:1 stratification based upon parent age, child gender and parental depression scores as self‐reported using the Patient Health Questionnaire (PHQ‐9; Kroenke, Spitzer, and Williams 2001). Further details regarding specific intervention provisions are provided by Bywater et al. (2018). All data was collected by self‐report, facilitated by a researcher in the family home, at four timepoints over a period of 18 months. Resulting data from the PSoC, in addition to demographic data, were extracted from the trial dataset for use in the current study.

## 3 Measures

### 3.1 Demographic Information

Demographic information was collected from each mother during an initial semistructured interview at the recruitment visit; this included the family's ethnicity, religion, family size, housing status and overcrowding, income level and employment status.

### 3.2 PSoC

The PSoC (Gibaud‐Wallston and Wandersman 1978; Johnston and Mash 1989) is a self‐administered questionnaire that contains 17 items developed to assess parenting self‐efficacy. It takes approximately 10 min to complete, and items are rated on a 6‐point Likert scale (1 = *strongly agree*, 6 = *strongly disagree*). In the two‐factor model, scoring for Items 2, 3, 4, 5, 8, 9, 12, 14 and 16 is reversed and comprises the satisfaction subscale. Scores for Items 1, 6, 7, 10, 11, 13, 15 and 17 comprise the efficacy subscale and remain unchanged. In the three‐factor version model, Items 14 and 12 form the interest scale, with the other items remaining on the satisfaction and efficacy scale. The scores are then summed to obtain a total score ranging from 17 to 102. A higher overall score indicates greater competence.

### 3.3 Data Analyses

We conducted confirmatory factor analysis (CFA) with the first timepoint of the data (*n* = 536), testing both the two‐ and three‐factor models. The diagonally weighted least squares estimator was used to account for polychoric correlations (where item responses are ordinal). For comparison of model fit between the two‐ and three‐factor models, we evaluated the comparative fit index (CFI; values > 0.90 are acceptable), root mean square error of approximation (RMSEA; values < 0.07 are acceptable) and standardised root mean square residual (SRMR; values < 0.08 are acceptable) (Hooper, Coughlan, and Mullen 2008).

After selecting the best fitting model, we tested the measurement invariance of that model with the first (baseline) and last timepoints of the data (18 months postbaseline). Measurement invariance establishes whether a measurement tool measures the same concept in the same way across time (Putnick and Bornstein 2016). Importantly, measurement equivalence does not mean there are no differences between the populations regarding a measured construct but establishes that respondents from different groups that have the same position on a trait of interest should provide a similar response (Davidov et al. 2014). We tested all three types of measurement invariance.

- Configural invariance means that the same latent variables are measured by the same items in all groups in the same arrangement and is used as a baseline for further invariance testing.
- Metric invariance additionally requires that all loadings of items are the same across groups. If metric invariance is supported, we can conclude that the groups are interpreting the items in the same way, and if it is not supported, the measurement invariance may imply that some items are more important to the construct for one group than for the other.
- Scalar invariance implies that both factor loadings and indicator intercepts are the same across groups, allowing meaningful comparison of latent means across all groups. If scalar invariance is supported, we can conclude that the two groups use the response scale in a similar way; if it is not supported, the invariance may imply systematic differences in the average item responses between groups that are not due to differences in the mean level of latent variables.

Each of the increasingly constrained invariance models is nested within the previous models; hence, we compared the previously described fit indices between the configural, metric and scalar invariance models, as well as the sample size‐adjusted Bayesian information criterion (BIC; values above 2 indicate that the evidence against the other model is positive).

### 3.4 Software

Descriptive statistics were used to summarise sample characteristics in Stata Version 18, and CFA and measurement invariance testing used *lavaan* in RStudio (Rosseel 2012).

## 4 Results

### 4.1 Research Question 1

**FIGURE 1:**
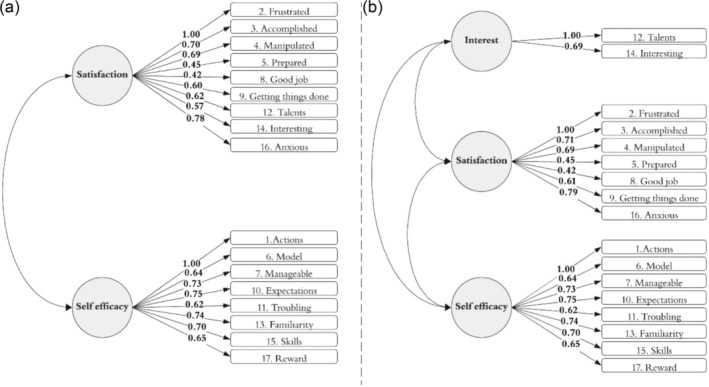
(a, b) Model structure and standardised factor loadings for two‐factor and three‐factor models at baseline.

Figure 1a,b presents the model structure and loadings for both models at baseline (i.e., the first timepoint, when children are aged birth—8 weeks old). All factor loadings were above 0.3, and all items were found to significantly load onto the latent constructs at both timepoints (*p* < 0.05).

Table 2 presents model fit values for both models. Both CFI values were within the acceptable ranges, and values for Model 2 were more favourable (as higher values indicate a better fit). RMSEA and SRMR values were also within acceptable ranges, and values for Model 2 were more favourable (as lower values indicate better fit). We therefore tested the longitudinal measurement invariance of the three‐factor model.

**TABLE 2:** Model fit values for two‐ and three‐factor models at baseline.

	Model 1 (two factors)	Model 2 (three factors)
CFI	0.984	0.986
RMSEA [95% CI]	0.055 [0.048–0.063]	0.052 [0.044–0.060]
SRMR	0.058	0.056

### 4.2 Research Question 2

Table 3 and Table S1 show that tests of configural invariance indicated values within the acceptable range, meaning that configural variance was met. The tests of metric invariance indicated worse model fit, with both CFI and SRMR outside of the acceptable range of values. The tests of scalar invariance again indicated a worse model fit, with both CFI and SRMR outside of the acceptable range of values.

**FIGURE 2:**
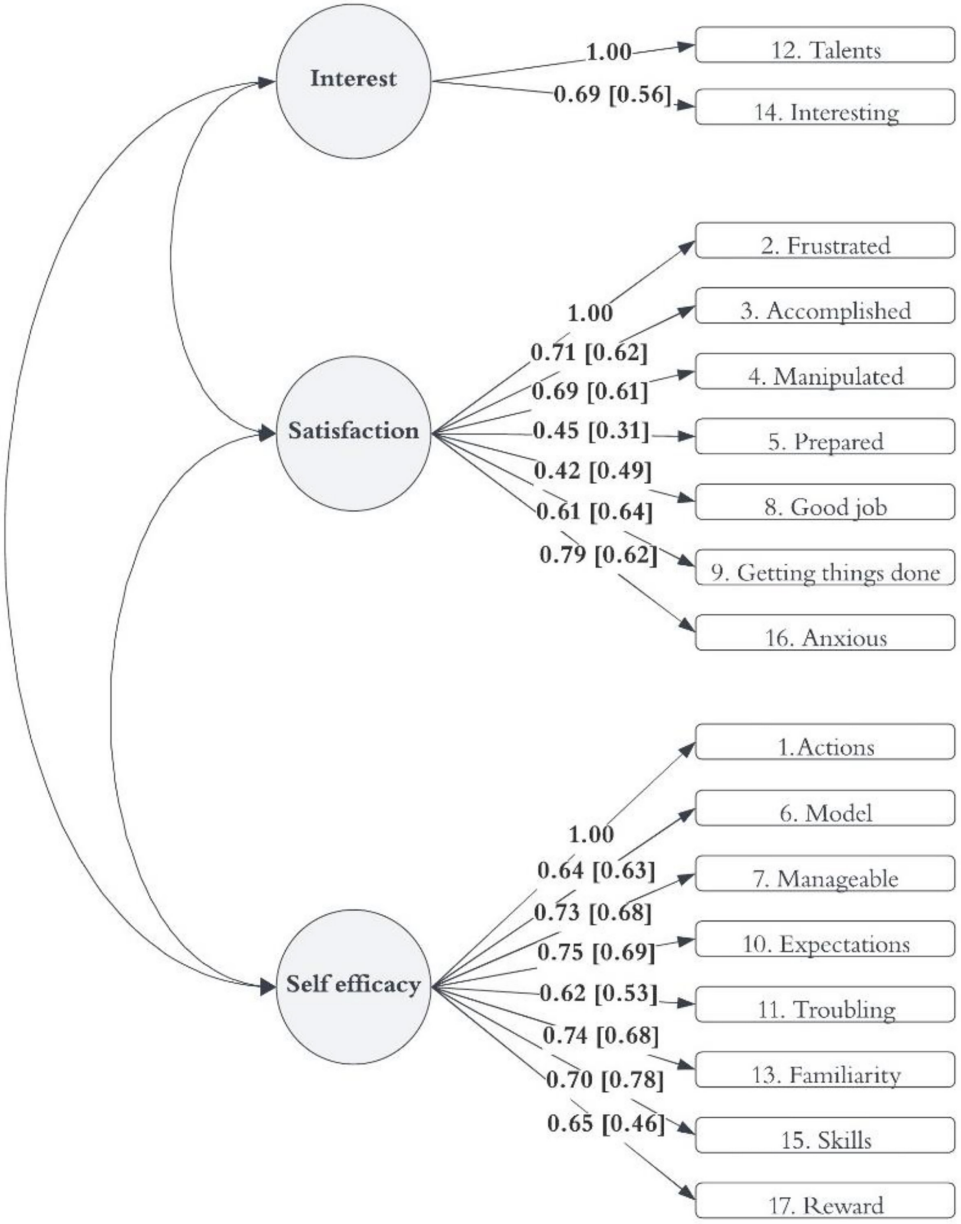
Standardised factor loadings for three‐factor model at baseline (factor loadings from 18‐month timepoint in square brackets).

We compared the factor loadings between the three‐factor model at baseline and at the 18‐month timepoint to investigate which items may have caused issues with metric and scalar invariance. Figure 2 and Figure S1 reveal that all items had lower factor loadings at the 18‐month timepoint, with the exception of Items 8 (“a difficult problem in being a parent is not knowing whether you're doing a good job or a bad one”) and 9 (“sometimes I feel like I'm not getting anything done”). Focusing on those with ≥ 10 point difference in factor loadings, we observe that the following items have lower loadings at 18 months:

- interest factor, Item 14 (“if being a mother/father of a child were only more interesting, I would be motivated to do a better job as a parent”).
- Satisfaction factor, Items 3 (“I go to bed the same way I wake up in the morning, feeling like I have not accomplished a whole lot”), 5 (“my mother/father was better prepared to be a good mother/father than I am”) and 16 (“being a parent makes me tense and anxious”).
- Efficacy factor, Item 17 (“being a good mother/father is a reward in itself”).

**TABLE 3:** Model fit values for tests of configural, metric and scalar invariance within the three‐factor model at baseline and 18‐month timepoint.

	CFI	RMSEA	SRMR	BIC
Configural invariance	0.926	0.041	0.051	42583.912
Metric invariance	0.859	0.055	0.504	42906.214
Scalar invariance	0.813	0.063	0.503	43123.388

## 5 Discussion

The current study sought to explore the longitudinal measurement invariance of the 17‐item PSoC using a sample of parents recruited to an RCT evaluation of a universal proportionate model of parenting support. Both a two‐ and three‐factor model demonstrated acceptable fit values, with the three‐factor model values being more favourable. The stability of this model was assessed with CFA. Analysis indicated that while configural variance was consistent across time, metric and scalar invariance standards were not met. The results suggest that the variance of the three‐factor model, consisting of the subscales interest, satisfaction and efficacy, is unstable over time for parents of children aged birth to 20 months. Based on these findings, we encourage researchers and clinicians to take care when using and interpreting results from the 17‐item PSoC with parents of very young children.

Our initial analysis identified that the three‐factor model was more favourable for explaining the structure of the PSoC items compared to the two‐factor model. This supports evidence from Gilmore and Cuskelly (2009) and Rogers and Matthews (2004), who have suggested the two‐factor model may be inadequate in comparison. The third factor had been previously described as *interest* containing Items 12 and 14, with Gilmore and Cuskelly (2009) reporting the addition of Item 17. Within our dataset, this factor only included Items 12 and 14. By their very nature, these items require a parent to reflect on their overall interest in their parenting role in accordance with their child's developmental stage. Previous research has generally utilised samples of parents of school‐aged children or has been cross‐sectional in nature, thereby rendering change over time obsolete. The current study uses data collected from a sample of parents of young infants over the course of the child's first 2 years of life—the same population that the PSoC was originally designed for. As a result, this is the first time that the interest subscale has been evidenced as a stable factor for this population.

Our analyses indicated that while the standards for configural invariance were met, that is, the number of factors and pattern loadings were consistent across time, both the metric and scalar invariance standards were not. The results therefore suggest that some items were more important for one construct at one timepoint in comparison to another. Furthermore, the loadings and indicator intercepts do not appear to be consistent across time. This further implies that there are systematic differences in average item responses over time that are not due to the differences in the mean level of latent variables. While we should note that some have debated the importance of scalar invariance, arguing that it is an unrealistic ideal and finding true scalar invariance with these strict tests of invariance is very difficult (Davidov et al. 2014), we deem this to be an important finding. Specifically, because more recent revisions and psychometric testing of the PSoC have indicated item response differences between groups of parents with children at four different life stages, that is, 0–3, 4–7, 8–12 and 13+ years (Gilmore and Cuskelly 2024).

While previous research has suggested that the wording of items influences responses, we have interpreted our findings to suggest that, even within a short period of development (i.e., birth—24 months), which is arguably the most rapid, our population demonstrates genuine shifts in how they may reflect on their parenting abilities considering changes in their children's ability and their relationship. This response shift pattern is considered to reflect genuine changes in an individual's meaning of a target construct over time (Sprangers and Schwartz 1999). It is proposed that changes in an individual's response to a self‐reported measure reflect three cognitive adaptational processes that occur organically in relation to significant changes in life circumstances. In the case of the present study, the changes in response to the PSoC reflect the adjustment and adaptations required following the birth of a child. These adaptations are suggested to comprise the recalibration of an individual's internal standards (or self‐awareness), the reprioritisation and change in personal values and beliefs and the reconceptualization or a change in the definition of what it means to be a parent. Sprangers and Schwartz (1999) previously suggested that response shift patterns are likely moderated by sociodemographic variables but also interact with coping styles to create direct and reciprocal feedback loops. As the majority of research on this phenomenon has focused on quality of life measures, further research is therefore needed to better understand how item responses on self‐reported measures of parenting, such as the PSoC, may change in response to age‐related developmental changes, which bring about different parenting challenges, and the subsequent shifts in the dyadic relationship over time.

The main strength of this research is that it is the only known study to explore the factor structure of the PSoC over time within the same population, specifically across a critical period in a child's life when risks for poor maternal mental health are greater, and the dynamics in the parent–child relationship are known to have long‐lasting effects on the future development of the child. Given the popularity of the PSoC in parenting research, particularly as an outcome measure in the evaluation of parenting programmes, the results from the three‐factor CFA provide initial evidence to suggest that this measure may not be a reliable measure of the construct ‘parenting competence’ for parents of young infants. As such, the results from this study should stimulate further discussion regarding the measure's validity and provoke further investigation of its appropriateness for use as a measure of change.

The main limitation of the study is that the current sample is drawn from a larger population of parents who participated in an RCT evaluation, which sought to test a universal proportionate model of parenting support based on a family's level of need. As such, the eligibility criteria were broad to encourage a representative sample of parents living in the targeted settings to participate. While the intervention was shown to be ineffective, comparisons to population norms indicate that the trial sample was not particularly representative of local or national samples in terms of demographic characteristics. Moreover, the current sample differed significantly from those excluded from the analysis due to missing data on marital status and educational qualifications. Therefore, the results may not be representative of the population from which the sample was drawn—parents with greater levels of need or more variable backgrounds. Further examination of the PSoC along different respondent demographic factors is required before we can make any meaningful inferences about its utility.

The findings have implications for both practice and research. While the PSoC is a commonly used measure of parenting self‐efficacy, our findings suggest little evidence to support its continued use with parents of young children without further exploration/validation. Further investigation of its structural validity, considering its sensitivity to developmental changes and subsequent shifts in parental perceptions of their own abilities, is warranted. Further work is also needed to establish the invariance of the measure across different groups. Researchers using the PSoC for cross‐sectional purposes should also strive to assess the underlying factor structure with their own samples before reporting findings according to the two‐factor model. We conclude that the continued use of the PSoC in research, specifically as an outcome measure to assess change over time, should be taken with care, as the presented evidence suggests it has little stability over time.

## Author Contributions

**Nicole Gridley:** conceptualization, investigation, writing – original draft, methodology, writing – review and editing, formal analysis. **Kate Mooney:** methodology, formal analysis, writing – review and editing, writing – original draft. **Sarah Blower:** conceptualization, investigation, writing – original draft, writing – review and editing, methodology. **G.J. Melendez‐Torres:** conceptualization, formal analysis, writing – review and editing, methodology, writing – original draft. **Vashti Berry:** conceptualization, methodology, writing – review and editing, writing – original draft. **Tracey Bywater:** funding acquisition, conceptualization, writing – original draft, writing – review and editing, methodology.

## Disclosure

We confirm that this manuscript has not and is not under consideration for publication elsewhere. The views expressed are those of the authors and not necessarily those of the NHS, the NIHR or the Department of Health and Social Care.

## Ethics Statement

Ethical approvals for the pilot RCT were granted by the University of York Education Ethics Committee (ref: FC15/03), and UK NHS REC 5 (ref: 15/WA/0178). Protocol No. Version 9, 26th February, 2018.

## Conflicts of Interest

The authors declare no conflicts of interest.

## Supporting information


**Table S1** *.* Model fit values for tests of configural, metric and scalar invariance within the two‐factor model.
**Figure S1.** Standardised factor loadings for the two‐factor model at baseline (factor loadings from 18‐month timepoint in square brackets).

## Data Availability

The data that support the findings of this study are available from the corresponding author upon reasonable request.
