# Supplementary material for: Longitudinal Measurement Invariance of the Parenting Sense of Competence (PSoC): Evidence to Question Its Use?
Source: Child Care Health Dev. 2024 Dec 30;51(1):e70030. doi: 10.1111/cch.70030 (PMC11684975; doi:10.1111/cch.70030)
Supplement: Supplementary file 1 — Table S1 . Model fit values for tests of configural, metric and scalar invariance within the two‐factor model. Figure S1. Standardised factor loadings for the two‐factor model at baseline (factor loadings from 18‐month timepoint in square brackets). [file CCH-51-e70030-s001.docx]

***Supplementary materials: longitudinal measurement invariance for Model 1 (two factors)***

*Table S1.*

Model fit values for tests of configural, metric, and scalar invariance within the two-factor model

|  | **CFI** | **RMSEA** | **SRMR** |
| --- | --- | --- | --- |
| **Configural invariance** | 0.917 | 0.043 | 0.052 |
| **Metric invariance** | 0.855 | 0.056 | 0.437 |
| **Scalar invariance** | 0.809 | 0.063 | 0.437 |

**
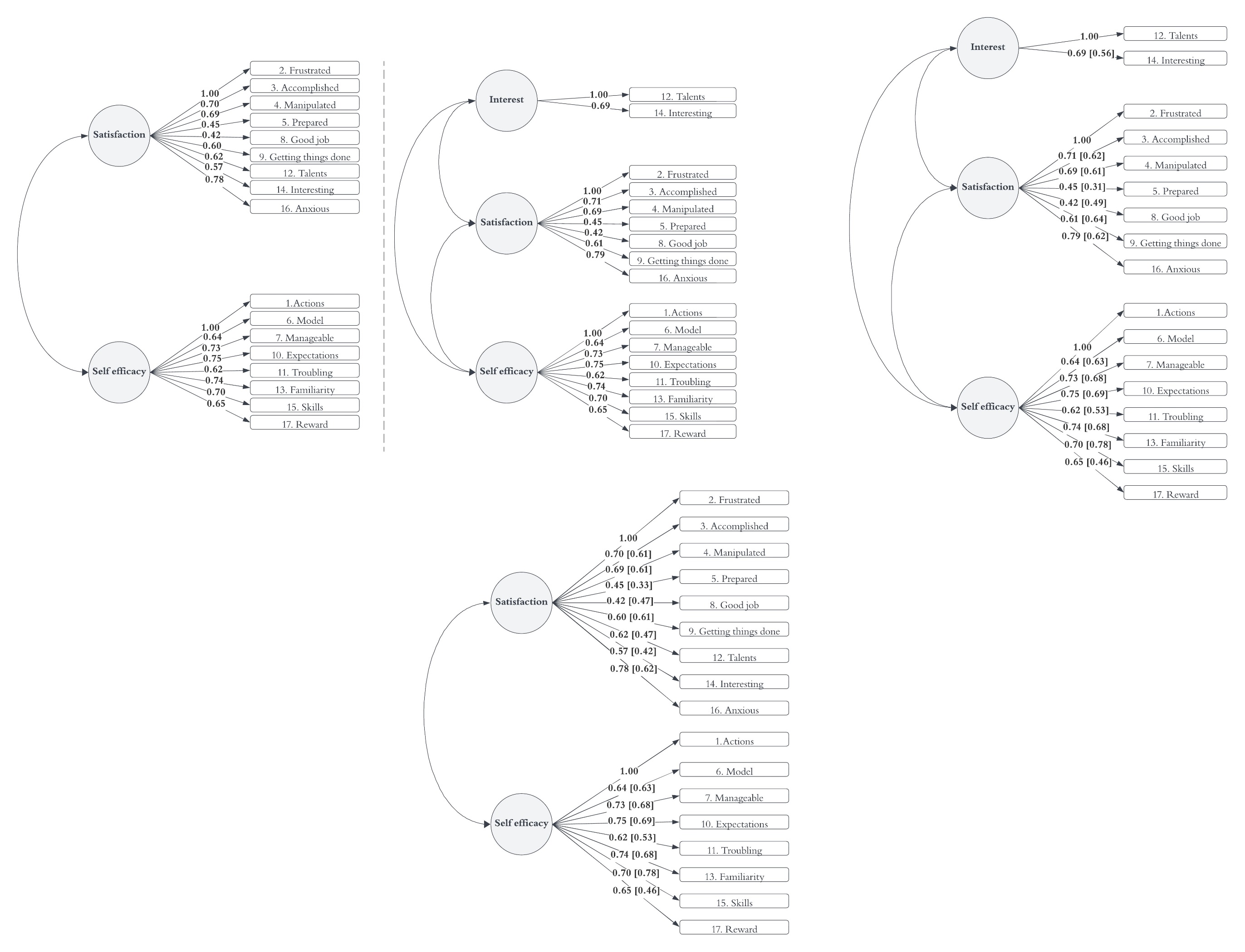
**

Figure S1. Standardised factor loadings for two-factor model at baseline [factor loadings from 18-month timepoint in square brackets]
